# Levels of 4-(methylnitrosamino)-1-(3-pyridyl)-1-butanone (NNK) in raw wastewater as an innovative perspective for investigating population-wide exposure to third-hand smoke

**DOI:** 10.1038/s41598-018-31324-6

**Published:** 2018-09-05

**Authors:** Foon Yin Lai, Katerina Lympousi, Frederic Been, Lisa Benaglia, Robin Udrisard, Olivier Delémont, Pierre Esseiva, Nikolaos S. Thomaidis, Adrian Covaci, Alexander L. N. van Nuijs

**Affiliations:** 1 Toxicological Centre, Department of Pharmaceutical Sciences, University of Antwerp, Universiteitsplein 1, 2610 Antwerp, Belgium; 2 Laboratory of Analytical Chemistry, Department of Chemistry, National and Kapodistrian University of Athens, Panepistimiopolis Zografou, 15771 Athens, Greece; 3 Ecole des Sciences Criminelles, University of Lausanne, 1015 Lausanne, Switzerland

## Abstract

Tobacco smoking is the major cause of many chronic diseases, especially lung cancer. Knowledge about population-wide tobacco use and exposure is essential to characterise its burden on public health and evaluate policy efficacy. Obtaining such knowledge remains challenging with current methods (e.g., surveys, biomonitoring) but can be achievable with wastewater analysis, a promising tool of retrieving epidemiology information. This study examined population-wide exposure to tobacco toxicants and carcinogens through wastewater analysis and explored relationships among these chemicals. Cotinine, *trans*-3′-hydroxycotinine, anabasine, anatabine and 4-(methylnitrosamino)-1-(3-pyridyl)-1-butanone (NNK) were analysed in samples from Greece, Switzerland and Belgium, where tobacco control policies are different. Measured per-capita mass loads were ranked as: nicotine biomarkers ≫ tobacco markers > carcinogens. Relationships between nicotine biomarkers and tobacco markers implied substantial use of non-tobacco nicotine items besides tobacco products. Geographic profiles of tobacco markers revealed higher levels in Geneva and Athens than Geraardsbergen and Ninove. Environmental third-hand smoke led to NNK detection, with elevated levels observed in Athens where indoor smoking is widespread, posing potential health risks to the population. Our novel outcomes are relevant for public health authorities as they provide indications about external exposure and can thus be used to plan and evaluate tobacco control policies.

## Introduction

Worldwide, tobacco smoking remains a priority public health concern due to its negative impacts on health, from single individuals to entire populations^1,2^. Approximately 22% of all cancer deaths were attributed to tobacco use, particularly lung cancer^3,4^. Globally, tobacco smoking causes more than 6 million deaths per year^3,4^. This figure has been predicted to rise to 8.3 million by 2030^5^. Since 1950s, numerous studies have shown the adverse effects on human health from the intake of toxicants and carcinogens in first-hand and second-hand tobacco smoke^6,7^. In contrast, exposure to these chemicals through third-hand smoke (THS) started raising concern only about a decade ago^6,7^.

Third-hand smoke, a newly recognised form of environmental exposure to tobacco-related chemicals, is the deposition of toxicants and carcinogens from second-hand smoke on indoor surfaces and objects (e.g., walls, ceilings, carpets, clothing and furniture)^6–8^. Third-hand smoke also coats on body surfaces (e.g., skin and hair) and clothing fabrics of both smokers and bystanders. Residual nicotine from THS accumulated on surfaces over time has been showed to react with ambient nitrous acid to form tobacco-specific nitrosamines (TSNAs)^9^, especially 4-(methylnitrosamino)-1-(3-pyridinyl)-1-butanone (NNK), a Group I carcinogen classified by the International Agency for Research on Cancer (IARC). Recent studies have also detected nicotine and NNK in indoor dust and found these chemicals in indoor environments due to THS contamination (e.g., cars and homes of smokers and non-smokers)^10–12^. These chemicals attributed to THS have even been noticed in hospital settings^13^. Exposure pathways for THS include inhalation (e.g. dusts) and dermal uptake from contact with contaminated surfaces and clothes/textiles, as well as ingestion from the hands or food that are exposed to tobacco smoke^7^. Negative health impacts of THS exposure on rodents have been recently revealed^8,14^; however, further studies are required to assess health risks for humans.

So far, environmental sampling methods to estimate the extent of indoor THS exposure have mainly relied on the collection of indoor air, dust and surface-wipe samples^7,11–13,15^. These sampling strategies however may remain less practical as they are labour-intensive, especially when it comes to large scale monitoring studies over the whole population, and can be confronted with ethical or privacy issues (e.g., sampling in individual households). Furthermore, people are mostly unaware of being exposed to THS as it clings to surfaces long after smoking events. Measurements of THS-related compounds in the environment can be used to assess external exposure to THS. Collectively, these aspects, together with the fact that THS is pervasive, suggest that there is a clear need for innovative and efficient approaches which can complement existing methods used to evaluate, over space and time, environmental exposure to THS in large populations.

Raw wastewater has been recognised as an emerging and useful source to retrieve epidemiological information about substance use and exposure to environmental chemicals at the population level^16^. The approach relies on the fact that wastewater generated by the population of a given catchment contains biomarkers of lifestyle, health and exposure to environmental pollutants. These biomarkers are collected in the sewage system and conveyed to municipal wastewater treatment plants (WWTPs). For instance, studies have measured endogenous chemicals in raw wastewater collected at the inflow of WWTPs, such as isoprostanes (oxidative stress markers), to assess the potential health status in the populations^17^. Also, analysis of relevant (bio)markers in raw wastewater has been illustrated as an alternative tool to monitor community-wide exposure to environmental chemicals, such as pesticides, phthalate plasticisers and organophosphorus flame retardants^18–21^. Analogously, the concept can be extended to investigate population-wide exposure to environmental THS, where levels of chemicals related to THS in wastewater originate from, such as, textiles (e.g., laundries), washing off indoor surfaces (e.g. cleaning), human body (e.g., hair and hand washing), etc.

The advantages of wastewater analysis can be also seen through the numerous studies^22–24^ that have measured nicotine biomarkers (i.e., cotinine and *trans*-3′-hydroxycotinine) in raw wastewater to back-estimate nicotine-equivalent cigarette use in communities. Despite limited studies, other minor tobacco alkaloids (i.e., anabasine and anatabine) in wastewater were suggested to reveal population tobacco use^25^. To date, no attention has been drawn to the potential co-occurrence of nicotine biomarkers, tobacco alkaloids and carcinogens (e.g., NNK) through wastewater analysis. This could provide highly valuable information on the extent of population-wide exposure to the carcinogens from tobacco smoking. Also, a potential emphasis can be on environmental THS since NNK is present in tobacco smoke and showed as a key THS compound^7,10,12,26^, while anabasine and anatabine are specific to tobacco use^27^. Furthermore, the relationship between nicotine biomarkers and tobacco specific alkaloids in wastewater may provide indications about the prevalence of the use of non-tobacco nicotine items (e.g. electronic cigarettes, nicotine replacement products) in populations. To the best of our knowledge, these last three aspects have not been studied so far.

By means of wastewater analysis, the overall objective of our study was to explore population-wide exposure to the tobacco-related (bio)markers across a selection of cities in three European countries (Belgium, Greece and Switzerland) where have different tobacco control policies^28,29^. To achieve the goal, raw wastewater samples (daily composite) were collected at the inflow of municipal WWTPs and analysed for cotinine, *trans*-3′-hydroxycotinine, anabasine, anatabine and NNK based on our previously validated analytical method^30^. Specifically, this study aimed to: (a) assess the geographical level and profile of these chemicals over the three different European populations; (b) evaluate the relationship between nicotine biomarkers and tobacco markers for a possible indication of nicotine use from non-tobacco nicotine items apart from tobacco products in the populations; and (c) examine wastewater levels of NNK in relation to population-wide external exposure to THS, and explore its association with tobacco markers for a model that allows preliminary evaluations of population-wide exposure to NNK in THS from tobacco use.

## Results

### Geographical patterns and levels of target (bio)markers across different locations

The target (bio)markers were measurable in all samples with the levels in a decreasing ranking: nicotine biomarkers (cotinine: 1450–6360, 2550 (median) ng/L; *trans*-3′-hydroxycotinine: 3130–10900, 4960 ng/L) ≫ tobacco markers (anabasine: 11–38, 19 ng/L; anatabine: 26–69, 40 ng/L) > carcinogens (NNK: 1.6–8.8, 2.3 ng/L). The ranking pattern was the same across all locations.

**Figure 1:**
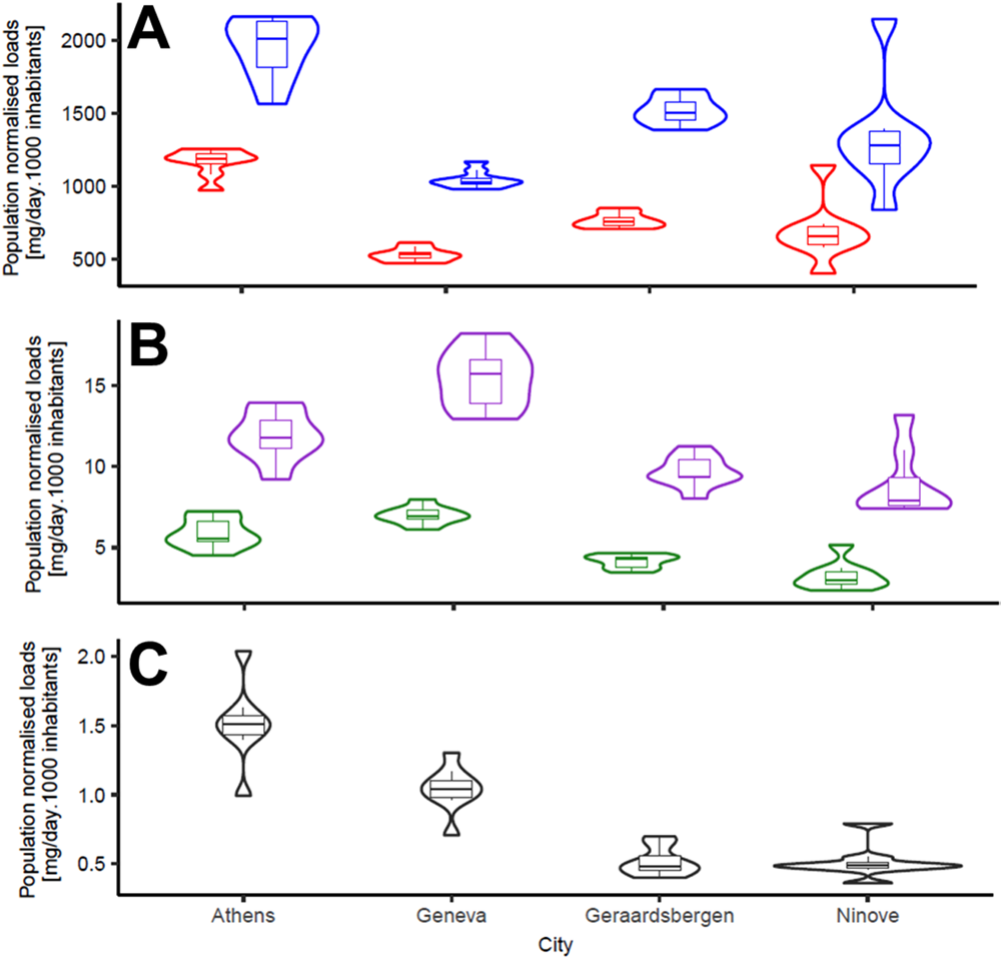
Violin plots of population-normalised mass loads (mg/day/1000 inhabitants) for (**A**) cotinine (red) and *trans*-3′-hydroxycotinine (blue); (**B**) anabasine (green) and anatabine (purple); and (**C**) NNK in different studied locations. Each violin plot includes a boxplot which indicates the median, inter-quantile range and range of daily estimates over the monitored period.

To compare data across locations, population-normalised daily mass loads (mg/day/1000 people) (Fig. 1) were computed through Monte Carlo simulation using measured concentrations, daily wastewater flow data and catchment populations. Differences between locations were assessed statistically using Mann-Whitney test. The highest daily load (median) of cotinine and *trans*-3′-hydroxycotinine was observed in Athens (1160 and 1960 mg/day/1000 people respectively) followed by the two Belgian cities (Geraardsbergen: 740 and 1470 mg/day/1000 people; Ninove: 640 and 1250 mg/day/1000 people, respectively) (Athens *vs.* Belgian cities: *p* < 0.001) and Geneva (520 and 1010 mg/day/1000 people, respectively) (Athens *vs.* Geneva: *p* < 0.0001). For anabasine and anatabine, Geneva (6.7 and 15 mg/day/1000 people, respectively) showed the highest daily loads followed by Athens (5.4 and 11 mg/day/1000 people, respectively) (Geneva *vs*. Athens: *p* < 0.05, anabasine; *p* < 0.002, anatabine). Geraardsbergen and Ninove were found at the lowest daily loads of anabasine (4.2 and 2.9 mg/day/1000 people, respectively) and anatabine (9.1 and 7.7 mg/day/1000 people, respectively) (Geneva *vs*. Belgian cities: *p* < 0.0005). The largest daily loads of NNK were measured in Athens (1.5 mg/day/1000 people) followed by Geneva (1.0 mg/day/1000 people) (Athens *vs*. Geneva: *p* < 0.005), whereas lowest daily loads were found in the two Belgian cities (0.5 mg/day/1000 people; Athens *vs*. Belgian cities: *p* < 0.0002).

Mann-Whitney analysis did not show any distinct weekly pattern, suggesting that day-to-day loads of target (bio)markers were similar over the monitoring period across different locations. The load of each chemical on an individual day was approximately 12% (median) of the total weekly load (11–13% during weekdays and 10–13% on weekends).

### Relationships among target (bio)markers

**Figure 2:**
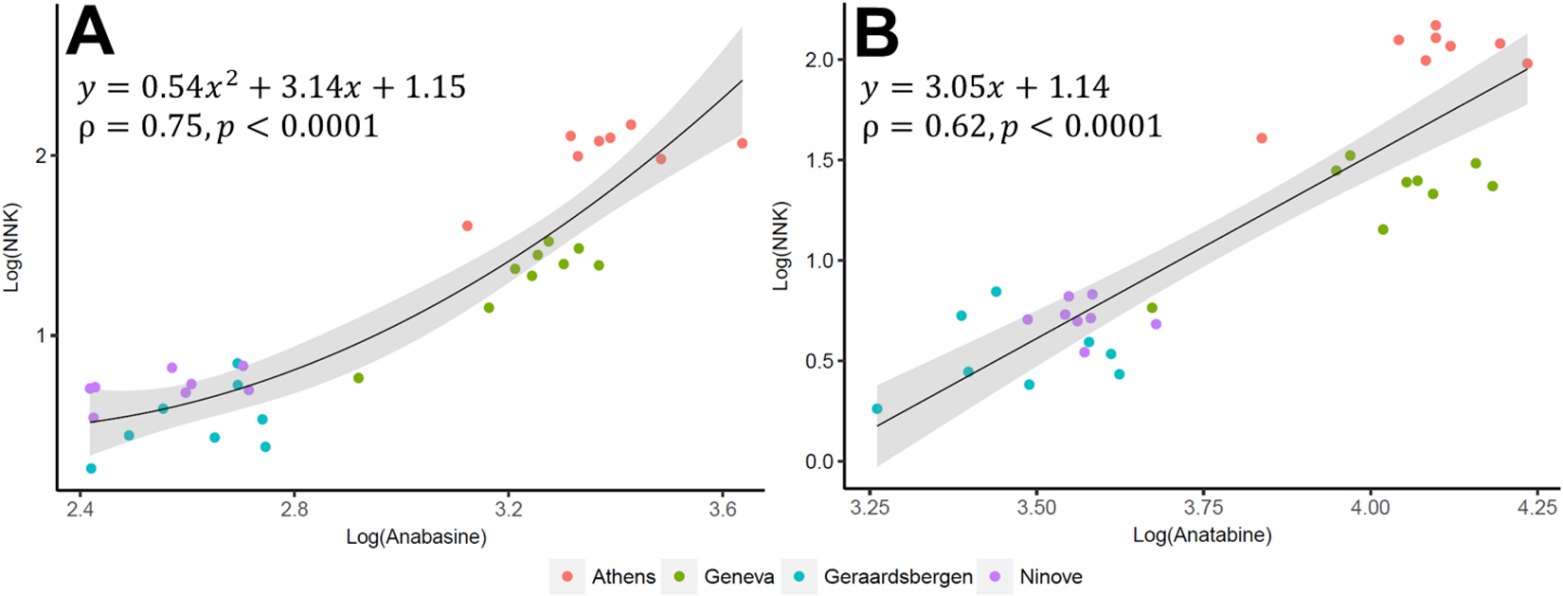
The best fit model for the relationship of (**A**) NNK *vs*. anabasine and (**B**) NNK *vs*. anatabine based on log-transformed population-normalised mass loads (mg/day/1000 inhabitants) in the studied populations.

According to correlation analysis, the target (bio)markers demonstrated different relationships. A strong correlation (Spearman ρ = 0.95, *p* < 0.0001) was found between cotinine and *trans*-3′-hydroxycotinine in wastewater of all locations, in agreement with findings from previous studies^22,31^. Similarly, the relationship between anabasine and anatabine was statistically significant (ρ = 0.90, *p* < 0.0001). However, no significant correlations were noticed between nicotine biomarkers (cotinine, *trans*-3′-hydroxycotinine) and tobacco alkaloids (anabasine and anatabine). NNK exhibited a strong association with anabasine (ρ = 0.75, *p* < 0.0001) and anatabine (ρ = 0.62, *p* < 0.0001) and a weak, but statistically significant, correlation with cotinine (ρ = 0.38; *p* = 0.03) and *trans*-3′-hydroxycotinine (ρ = 0.35; *p* = 0.05). The relationship between NNK and the two tobacco alkaloids was further assessed using a generalised linear model (Fig. 2). This was performed using log-transformed population-normalised mass loads with NNK as the dependent variable and anabasine or anatabine as the independent variable (Fig. 2). The best model, showing the smallest mean square error (MSE), was selected and its performance was assessed using leave-one-out cross-validation. A quadratic function was found to be the best model for NNK *vs*. anabasine while a linear function performed the best for NNK *vs*. anatabine. The obtained models can assist in estimating levels of NNK based on levels of anatabine and anabasine measured in wastewater in future studies. Particularly, anabasine appeared to be a better predictor of NNK compared to anatabine (i.e., Akaike information criterion: 2.63 *vs*. 14.5 and MSE: 0.065 *vs*. 0.085, respectively). The developed model is supposed to provide only indicative and preliminary information about levels of NNK in wastewater based on those of anabasine and anatabine since NNK has not been to date among the commonly monitored compounds while anabasine and anatabine levels have been previously reported^25^.

## Discussion

The present article explored the co-occurrence of toxicants and carcinogens associated with tobacco use (especially smoking) at the population level using wastewater as an information source. Through a selection of cities from three European countries with different prevalence of tobacco use and tobacco control policies (e.g., indoor smoke-free environments, restrictions on advertising tobacco products), this study exhibits new findings and perspectives from which the prevalence and public health related to population tobacco smoking can be assessed.

The strong and significant correlation between cotinine and *trans*-3′-hydroxycotinine suggested a substantial consumption of and exposure to nicotine in the studied populations. This can be also revealed by examining the concentration ratio of these two chemicals in wastewater (*trans*-3′-hydroxycotinine/cotinine, average: 1.9 ± 0.2 (standard deviation, SD)), which is similar to the urinary excretion found in clinical studies^32,33^ (average: 2.1 ± 0.6 SD, n = 80) after nicotine intake. The occurrence of cotinine and *trans*-3′-hydroxycotinine in the samples could result not only from exposure of first- (i.e. consumption) and second-hand smoke to nicotine, but also THS. In this light, recent studies^11,34^ have shown that levels of nicotine in household dust were relatively higher compared to other tobacco alkaloids, as a result of tobacco smoking and also the use of non-tobacco nicotine items (e.g., electronic cigarettes). Indoor human exposure to nicotine through THS has been only recently reported^13,35^. Although being the most abundant alkaloid (about 95%) found in tobacco, levels of nicotine biomarkers measured in this study reflect the use of all kinds of nicotine-containing products, from tobacco products (e.g., cigarettes, cigars, snuss, chewing tobacco) to non-tobacco nicotine items (e.g., electronic cigarettes and products used for nicotine-replacement therapies, such as patches, gum, sprays), at the population level. With the increasing use of non-tobacco nicotine items, especially electronic cigarettes^36^, back-estimating population tobacco/cigarette use based on cotinine and *trans*-3′-hydroxycotinine, as earlier proposed^22^ and applied in previous studies^23,24,31^, will become less relevant for future studies. In fact, this may be readily revealed in our data as the two nicotine biomarkers and tobacco-specific alkaloids were not correlated with one another; yet, such correlation was observed in urine from smokers^27^ and these compounds were found stable in wastewater^25,30^. Despite of potential inter-individual variations, the overall excretion rate would tend to an average value when taking into account a large number of people, which is the case for WWTP catchments. The stability test conducted so far has shown that the target chemicals remained stable in raw wastewater^25,30^; but future studies with the presence of biofilms would be useful for a further evaluation. Overall, the non-correlation observed between the two nicotine biomarkers and tobacco-specific alkaloids suggested that the consumed nicotine over the studied populations could be sourced from the use of non-tobacco nicotine items in addition to tobacco products. Actually, the prevalence of the current use of electronic cigarettes or similar devices in Greece (3%) and Belgium (4%) was above the average prevalence of the 28 European member states (2%)^37^. In Switzerland, about 0.3% of the population has used electronic cigarettes daily^38^ and approximately 16% of the heavy smokers relied on non-tobacco nicotine items to stop smoking, with an increasing trend of electronic cigarettes as the first choice^39^.

Anabasine and anatabine have been reported in urine of smokers and used as specific markers for the detection of tobacco use in clinical and human biomonitoring studies (e.g^27,40^). Besides transferring to mainstream smoke, anabasine and anatabine could thermally decompose during tobacco burning^40,41^ and thus remain less available in second- and third-hand smoke. Since our data showed a ratio of these two markers (anatabine/anabasine, average: 2.2 ± 0.4 SD) consistent with previous clinical studies (average: 1.9 ± 0.6 SD, n = 111)^27,40^, as well as the significant correlation between their levels measured in wastewater, the detection of anabasine and anatabine in the samples was more likely attributed to tobacco use (i.e., first-hand smoke) in the studied populations. Furthermore, the ratio of anabasine to anatabine in this study was also similar to that measured in Australian wastewater samples^25^, implying a consistent observation.

There was a geographical difference in the levels of the two tobacco markers, reflecting distinctive prevalence of tobacco use among the studied locations. Significantly high levels of anatabine and anabasine measured in Athens and Geneva than Geraardsbergen and Ninove may suggest a more widespread use of tobacco in large cities than small towns. However, different tobacco control policies employed in the studied countries cannot be excluded for examining the observed geographic profile. Still, similar geographical findings where higher usages in large cities than small towns were also observed for other substances, such as alcohol^42^ and illicit drugs^43,44^, in different international studies based on wastewater analysis. Levels of anabasine and anatabine measured in the two small Belgian towns were very similar to those in the city of Adelaide, a large city in Australia^25^. This is in line with the latest figure from the World Health Organisation (WHO) about prevalence of tobacco use, which was substantially lower in Australia (15%) compared to most European countries (13–42%) in 2015^45^.

While the WHO data reported Greece (42%) as the country in Europe with the highest prevalence of tobacco smoking, the highest levels of anatabine and anabasine were measured in Geneva, not Athens. Yet, a very recent study conducted in Switzerland^46^ pointed out a likely underestimation of the actual prevalence of smoking in Switzerland since smokers tended to under- and/or mis-report their smoking habits in population-based surveys. The study also reported that Switzerland was ranked as the last European country regarding bans on tobacco advertising in public places (e.g. billboards, outdoor displays, etc.), magazines, newspapers and on the internet, which are considered by the WHO as one of the most effective ways to reduce tobacco consumption^47^. Our data may suggest that prevalence of tobacco use in Geneva could be higher compared to Athens, at least over the short period considered in our study. These findings highlight the high geographical dimension of population tobacco use and the utility of data comparison by means of wastewater analysis, allowing to prioritise cities and regions with interventions in the future.

The moderate to strong relationships (Fig. 2) between NNK and the two tobacco markers (anabasine and anatabine) indicate that the detection of NNK is linked to tobacco use. Since NNK could not be measured in urine of cigarette smokers^48^, first-hand smoking could not be the major source of NNK in wastewater. Second-hand smoke is also unlikely the main contributor of NNK to wastewater as it lasts over only short intervals^7^. It appears impossible to exclude THS as the predominant source of NNK in wastewater because of its environmental ubiquity and pervasiveness from tobacco smoking^6–8,10–12^. There are numerous routes for THS to be dispersed in the environment. For instance, recent studies have shown that despite smoking outside, hospitalised smokers and visitors re-entering a hospital facility caused indoor THS contamination of nicotine and NNK through their articles (e.g., clothes, hands and hair of both smokers and non-smokers) being contaminated with tobacco smoke^13,49^. This phenomenon was also reported in other settings, such as households, hotels, cars, casinos, etc.^7,15^. As THS can be readily dispersed and transported from outdoors to indoors, these previous studies generally suggest that exposure to tobacco alkaloids and carcinogens in the environment cannot be easily avoided, even with policies of banning indoor smoking. Obviously, higher levels of these compounds, particularly nicotine and NNK, can be reasonably expected in places where indoor tobacco smoking is widespread and/or where the policy of banning indoor smoking is not being strictly enforced. In such environments, THS will be much more prevalent and the measurement of NNK could be used as an indication of population-wide external exposure. Our data clearly reflects this phenomenon, with elevated levels of NNK and nicotine biomarkers observed in Athens, where the policy of prohibiting indoor smoking (e.g., cigarettes, cigars, electronic cigarettes, etc.) in public places (e.g., transports, offices/workplaces, universities, restaurants, café, pubs, etc.) is not strictly enforced, while this is not the case for the other studied locations^28,29^. This may pose higher health hazards to the Athenian population from being exposed to tobacco carcinogens indoors. Our finding agrees with the latest public health report of the European Commission in 2017 documented the assessment of exposure to tobacco smoke in public places^37^. Greece was reported as a stand out country where 87% and 78% of the respondents who encountered indoor smokers in bars and restaurants, respectively, in the last six months^37^. Our data highlight the potential of wastewater analysis to indirectly monitor environmental contamination of NNK from THS at the population level, as a complimentary tool to the existing methods, and also to evaluate the effectiveness of law enforcement and intervention strategies.

Being carcinogenic, it is highly compelling to assess NNK levels in wastewater over time for a continuous evaluation of potential health hazards to populations in relation to tobacco smoking. For such assessments, NNK concentrations could be directly measured in wastewater or estimated using the developed model (Fig. 2) with previous wastewater data where the ratio of anabasine to anatabine is consistent with results from urine analysis in clinical studies. This would allow to obtain an estimate of NNK levels based on concentrations of tobacco markers measured in wastewater for a preliminary indication of the extent of population-wide levels of carcinogens linked to THS. Beyond the fact that these chemicals have been measured in other studies, this also offers an analytical advantage since anabasine and anatabine are in substantially higher concentrations compared to NNK, and thus their determination is less challenging.

As with every monitoring approach, there are a few potential uncertainties which need to be taken into account when interpreting the results. First, chemical leaching from littered cigarette butts in surface runoff waters might lead to the occurrence and high level of tobacco-related chemicals (e.g. NNK) in wastewater during precipitation events (i.e. high wastewater flow). However, this is considered very limited due to an absence of positive correlations between concentrations of NNK and wastewater flows in the studied locations. This further implied that the key sources of NNK in wastewater were unlikely from environmental runoff waters and precipitations outdoor, but rather THS contamination (e.g. in textiles, indoor settings and human body being exposed to tobacco smoke) in the populations. Daily activities, for example laundries, indoor cleaning and washing of hands and hair, likely lead to the contribution of THS-related chemicals (e.g. NNK) measured in wastewater. Our data on the significant association of the levels between NNK and the tobacco markers reinforced that the presence of NNK in wastewater was highly likely due to events of tobacco smoking by humans. Second, the obtained data spans over a period of about one week and thus may not be representative of levels measured in other periods of the year, since seasonal variations in tobacco smoking could occur, as it is the case for illicit drugs^50,51^. Therefore, sampling over prolonged periods, as well as the inclusion of additional locations will certainly provide further insights into the spatio-temporal patterns of exposure to tobacco-related toxicants and carcinogens in future studies. Lastly, back-estimation of tobacco use based on its specific markers, anabasine and anatabine, remains difficult due to the lack of human pharmacokinetic data on these compounds. Only one study has reported the urinary excretion of anabasine (about 28%) based on a single subject^52^. The availability of human pharmacokinetic studies will allow the specific back-estimation of tobacco consumption at the population level, similar to the practice for illicit drugs^44,53^. These consumption data could also assist in estimating tobacco black markets in a country. Nevertheless, the lack of pharmacokinetic data does not prevent the use of mass loads of target chemicals from detecting temporal and geographical changes, which will still reflect both tobacco use and exposure to its related carcinogens in the investigated populations over space and time.

In conclusion, our work provides the first exploratory study, based on wastewater analysis, to assess the occurrence and relationship between tobacco-related toxicants and carcinogens in four European cities. Specifically, taking into account our findings, the following conclusions could be drawn:

- Being frequently detected in the samples, the target (bio)markers and carcinogen were ubiquitous in wastewater from the studied populations.
- Besides tobacco products, consumption of and exposure to non-tobacco nicotine items in the populations led to the occurrence of cotinine and *trans*-3′-hydroxycotinine in wastewater. This result could be further evaluated by including countries with high consumption of non-tobacco nicotine items, such as snuss in Nordic countries, in future studies.
- Geographic profiles of anabasine and anatabine indicated a higher level of tobacco markers in Geneva and Athens compared to Geraardsbergen and Ninove, exhibiting distinctive patterns of tobacco use across the studied locations.
- Environmental THS contributed to the detection of NNK in the wastewater samples. This underlined potential health risks for the populations. Athens (Greece), the only location in this study where indoor smoking is not strictly prohibited and thus widespread, showed the highest level of NNK and nicotine biomarkers, in agreement with their occurrence at high levels in THS^10,11,13^. These findings highlight the need for intervention policies aiming at reducing exposure to THS in the population. Future long-term wastewater monitoring campaigns, conducted before and after possible introduction of new policies on restricting indoor smoking in Athens, will be of importance to evaluate its efficacy over time.

Overall, wastewater analysis is a useful tool to monitor population-wide nicotine and tobacco use. Notably, it facilitates an initial examination of external exposure to tobacco carcinogens through THS at the population level. Considering that THS is a dimension of emerging concerns, wastewater analysis provides both a new perspective from which to evaluate the extent of the problem as well as an additional and relevant source of information about a newly recognised threat to human health.

## Methods

Caution: Adequate safety precautions should be taken as NNK is a Group I carcinogen classified by IARC.

### Wastewater sampling

Raw wastewater samples (24-hour composite; 9AM-9AM) were collected at the influent stream of the WWTPs from Athens (Greece), Geneva (Switzerland) and Geraardsbergen and Ninove (Belgium). Each studied municipality has only one WWTP. The study period was over approximately one week: 23–30 January 2017 in Athens, 6–11 February 2017 in Geneva, 11–18 March 2015 in Geraardsbergen, 25 March-1 April 2014 in Ninove. No particular events occurred in the studied catchments over the monitoring period, and thus no substantial changes in population sizes are expected. The sewer catchment of the selected WWTP covered about 3,700,000 persons in Athens, 600,000 persons in Geneva, 29,100 persons in Geraardsbergen, 36,200 persons in Ninove. Sampling over 24 hours was conducted under refrigeration using flow-proportional mode in Athens, volume-proportional mode in Geneva and time-proportional mode (every 10 min) in Geraardsbergen and Ninove. These sampling methods have been previously used to assess illicit drug use in the respective populations^54^. An aliquot (1 L) of composite samples was transferred to polypropylene bottles prewashed with ultrapure water and methanol, and immediately frozen at −20 °C until analysis. For Geraardsbergen and Ninove, the samples have been archived in small aliquots (100 mL), frozen at −20 °C, and without extensive freeze-and-thaw cycles to minimise issues with stability. While the target chemicals were found stable in raw wastewater at 4 °C and even 20 °C over 24 h^30^, future studies regarding the chemical stability at −20 °C and over long-term storage should be carried out. Nevertheless, based on our previous finding^30^, the target compounds can be assumed to remain stable during the storage at −20 °C. Furthermore, the fact that consistent chemical ratios (*trans*-3′-hydroxycotinine/cotinine and anatabine/anabasine) in wastewater and in clinical urinary analysis further supports that the investigated chemicals are stable during long-term storage.

### Chemical analysis

Target chemicals in the samples were analysed using a previously validated analytical assay^30^. Briefly, for the analysis of anabasine, anatabine and NNK, solid-phase extraction with Oasis^®^ MCX cartridges (60 mg, 3 cc) was applied as a clean-up step and also pre-concentration (a factor of 1000) of the target chemicals from the samples (100 mL) treated with enzymatic deconjugation. As high in concentrations, cotinine and *trans*-3′-hydroxycotinine in the samples were analysed by direct injection (i.e., no sample extraction required). Samples and final extracts were injected (2 µL) and analysed using liquid chromatography (1290 Infinity, Agilent, California, USA) coupled to tandem mass spectrometry (AB-SCIEX Q-TRAP^®^5500, Ontario, Canada). Separation of the target analytes was performed using a Waters^®^ Atlantis T3 column (150 × 2 mm, 3 μm, Waters, Zellik, Belgium) with the mobile phase of (A) ultrapure water and (b) methanol, each consisted of 0.1% formic acid and 5 mM ammonium formate. Method quantification limits were estimated based on a signal-to-noise ratio of 10 in wastewater samples: 120 ng/L for cotinine, 37 ng/L for *trans*-3′-hydroxycotinine, 3.5 ng/L for anabasine, 9.0 ng/L for anatabine, and 1.5 ng/L for NNK. Together with a six-point calibration curve (0.5–100 ng/mL), concentrations of the target chemicals in the samples and extracts were quantified through the isotope dilution approach. This used the corresponding mass-labelled chemical to correct the analyte concentration with the extraction recovery of the analyte and matrix effect during instrumental analysis. Blank samples of pure water and samples spiked with native standards of the corresponding analyte were included in the extraction batches as the quality assurance and control to ensure appropriate analytical processes. No contaminations were detected in the blank samples. The average matrix-spike recovery (relative standard deviation, in 3 different days) was 101% (2.0%) for cotinine, 90% (4.5%) for *trans*-3′-hydroxycotinine, 91.4% (1.0%) for anabasine, 99.0% (3.3%) for anatabine, 104% (5.2%) for NNK.

### Data processing and analysis

Population-normalised mass loads (mg/day/1000 people) of the target chemicals were necessary to compare data among locations. This was obtained through two main steps: (a) multiplying the measured concentration with the daily wastewater volume to obtain daily mass loads (mg/day) and then (b) normalised it to the catchment population size. The calculation was computed using Monte Carlo simulations considering the uncertainty (standard error %) associated with chemical analysis (10%), sampling (10%), flow measurement (20%) and population (15%), as following the practice discussed in the field of wastewater analysis^55–58^. Normal distribution (mean, square of standard error) was set for these uncertainty components in the stimulation. Correlations between chemicals were tested with Spearmen rank analysis. Mann-Whitney test was used to assess the significant difference in chemical levels between locations as well as between weekdays and weekends. Generalised linear models were used to predict NNK levels from measured concentrations of anatabine and anabasine. Prior to modelling, data was log-transformed to accommodate for lack of normality and heteroscedasticity. Different functions were tested, in particular polynomials functions (degree 1 to 10) as well as square root polynomials. The performance of the tested models was assessed using leave-one-out cross-validation. The best model was selected based on the smallest MSE. Monte Carlo simulations, statistical tests and generalised linear models were computed using R software.

## Data Availability

The datasets generated during and/or analysed during the current study are available from the corresponding author on reasonable request.
